# Central retinal vein occlusion in a patient with breast carcinoma

**DOI:** 10.3205/oc000093

**Published:** 2019-02-12

**Authors:** V. G. Madanagopalan, V. Paneer Selvam, N. V. Sarath Sivan, N. V. Govindaraju

**Affiliations:** 1 Arasan Eye Hospital, Erode, India

**Keywords:** breast carcinoma, central retinal vein occlusion, microcytic hypochromic anemia

## Abstract

**Purpose:** Systemic malignancies may have ocular manifestations in the form of metastatic tumors, carcinoma associated retinopathy or central retinal vein occlusion (CRVO). Although CRVO has been mentioned in association with renal, lung, prostatic and ovarian malignancies, the association of CRVO with breast carcinoma is unreported. We report a patient with one such rare association.

**Methods: **We describe a patient with breast carcinoma who was diagnosed to have CRVO. The history, ocular examination, retinal optical coherence tomography scan, ocular ultrasound scan, hematological profile, mammogram details and aspiration cytology description of the malignant breast lesion are reported in this article.

**Results: **The retina showed extensive hemorrhages and dilated retinal veins. Complete hematologic evaluation revealed the presence of microcytic hypochromic anemia and increased hematocrit. These changes are possibly related to the malignant disease and might contribute to the pathogenesis of CRVO.

**Coclusion: **This case report demonstrates the rare association between breast malignancy and CRVO.

## Introduction

Any systemic condition that causes altered blood flow, abnormal blood viscosity, abnormalities in blood coagulability or induces changes in blood vessel structure can lead to central retinal vein occlusion (CRVO) [1]. It stands to reason that diabetes mellitus, hypertension, cardiovascular disease, blood dyscrasias (leukemia, lymphoma and polycythemia), paraproteinemias, systemic vasculitis or autoimmune diseases are often associated with CRVO [2]. Besides these common associations, anemia and systemic cancers have shown to be independently associated with CRVO.

## Case description

**Figure 1:**
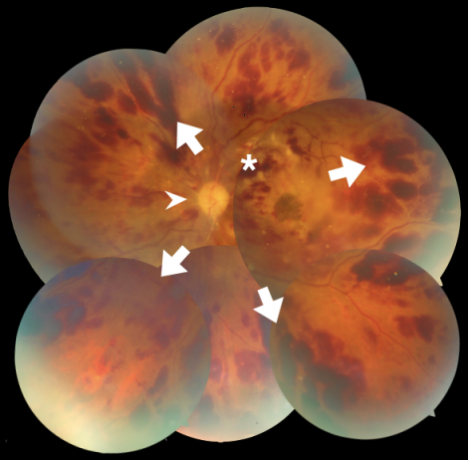
Montage image of the left retina shows optic disc pallor (arrowhead). Extensive superficial retinal hemorrhages are seen (arrows) and the retinal veins are dilated and tortuous consistent with central retinal vein occlusion. Few cotton wool spots are seen superotemporal to the optic disc (asterisk). Macular edema can also be made out.

**Figure 2:**
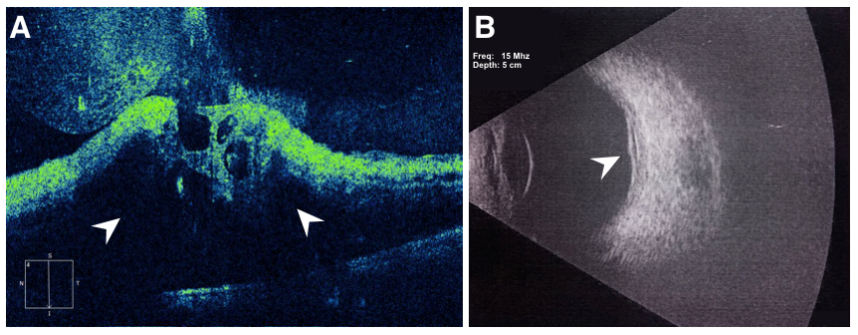
The optical coherence tomography image shows extensive macular edema seen as hyporeflective regions within the retina (arrowhead, 2A). Back shadowing due to superficial hemorrhages is also seen. Ultrasound imaging shows thickening of the retina over the posterior pole (arrowhead, 2B).

A 59-year-old female presented with decreased vision in the left eye (OS) for 15 days. She was recently diagnosed with left breast carcinoma. She had been advised mastectomy for the breast malignancy 1 month ago and had not followed up with her oncologist since then. The patient did not have diabetes, hypertension or hyperlipidemia. There was no history of coronary artery disease or stroke. The blood pressure recorded in the clinic was 128/90 mm Hg. Visual acuity in OS was 1/60 and VA in right eye (OD) was 6/6. Intraocular pressure in both eyes was 14 mm Hg by non-contact tonometry. The anterior segment examination in both eyes was normal and the pupil in OS did not show a relative afferent pupillary defect. Fundus exam of OS revealed mild pallor of the optic disc (arrowhead, Figure 1 (Fig. 1)). Multiple superficial and deep retinal hemorrhages were striking (arrows, Figure 1 (Fig. 1)). Few cotton wool spots were noted supero-temporal to the optic disc (asterisk, Figure 1 (Fig. 1)). Preretinal hemorrhage was present at the macula with macular edema. Retinal exam of the OD was normal. Optical coherence tomography of OS showed macular edema (arrowhead, Figure 2A (Fig. 2)) with a central foveal thickness of 1,162 microns (normal: 220–240 microns). Backshadowing was present due to retinal hemorrhages. Ultrasound scan of OS showed thickened retina at the posterior pole (arrowhead, Figure 2B (Fig. 2)) of the eye suggesting macular edema. There were no apparent ocular, optic nerve or orbital mass lesions.

**Figure 3:**
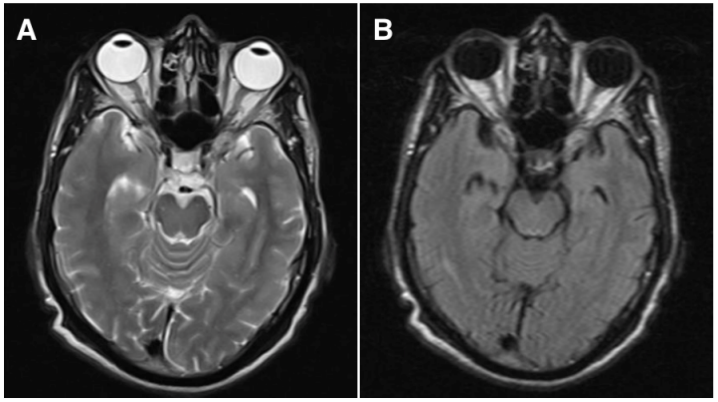
Magnetic resonance imaging of the brain and orbit by axial T2-weighted sequence (A) and fluid-attenuated recovery (FLAIR) sequence images (B) do not show any lesions in the choroid, optic nerve or orbit on both sides.

Hematological investigations showed that the hemoglobin was 9 gm% (normal: 12–15), platelet count was 255,000/mm^3^ (normal: 150,000–400,000), fasting plasma glucose was 106 mg% (normal: 80–120), blood urea was 34 mg% (normal: 15–40), serum Creatinine was 0.8 mg% (normal: 0.5–0.9), erythrocyte sedimentation rate was 28 mm at one hour (normal: 0–15), hematocrit was 89.8% (normal: 36–46), bleeding time was 2 minutes 05 seconds (normal: 0–7 minutes) and clotting time was 4 minutes 15 seconds (normal: 4–9 minutes). The lipid profile was normal. Peripheral blood smear showed microcytic hypochromic anemia with tear drop cells and Rouleaux formation. Total leucocyte count was normal with differential count showing eosinophilia (19%). The blood sample was negative for human immunodeficiency virus and hepatitis B virus. X-ray mammogram, sonomammogram and elastomammogram of the left breast in the craniocaudal and mediolateral oblique views showed a 2x 2.7 cm ill defined hypoechoeic and hypolucent lesion with irregular margins in the retro areolar region with architectural alterations and nipple retraction. The axillary lymph nodes were enlarged with the largest measuring 1x 0.9 cm. Ultrasound abdomen and magnetic resonance imaging of the head and orbit (axial T2-weighted sequence, Figure 3A (Fig. 3); fluid-attenuated inversion recovery sequence (FLAIR), Figure 3B (Fig. 3)) did not show any secondary mass lesions.

## Discussion

Of the many risk factors for CRVO, the metabolic syndrome (hyperglycemia, hypertension and hyperlipidemia) and past vascular events are considered most important [3]. In our patient, CRVO occurred in the absence of any of these risk factors. However, she had microcytic hypochromic anemia and breast carcinoma. The association of CRVO with anemia and few systemic carcinomas (renal, lung, prostate and ovarian carcinoma) has been described separately. In our patient, both anemia and carcinoma coexist and appear to contribute to the pathogenesis of CRVO. There are no previous reports of CRVO in association with breast carcinoma.

Cancer is a cause of hypercoagulability and an association of CRVO with systemic malignancies is well known [4], [5], [6], [7]. In malignancies, CRVO is considered a paraneoplastic process where dynamic interactions between the tumor cells, blood coagulation system, vascular endothelium, leucocytes and platelets contribute to the pathogenesis of CRVO. Rhone and colleagues have shown that the concentration of plasminogen activator inhibitor type 1 is increased and activity of tissue factor pathway inhibitor is reduced in patients with breast carcinoma [8]. These hematologic changes promote thrombosis. Therapeutic agents used in cancer treatment may also contribute to the process of CRVO [9], [10], [11]. Besides, when a metastatic tumor in the optic nerve causes compression, venous outflow channels are impeded and CRVO could occur [12]. The other rare paraneoplastic syndrome in carcinoma that can cause vision loss is cancer-associated retinopathy (CAR) [13], [14]. Unlike CRVO, which presents with striking retinal hemorrhages, the clinical picture in CAR is rather subtle. An immunological process directed at the tumor antigens is implicated in CAR. These antibodies cross-react with retinal antigens to produce symptoms that resemble those with retinitis pigmentosa.

Anemic retinopathy presents with extravasation of blood into the retina due to retinal vasodilation as a response to relative retinal ischemia in the setting of acute blood loss [15], [16]. Retinal hemorrhages in anemic retinopathy are usually located in the superficial retinal layers and are white centered. Occasionally, hemorrhages are also encountered anterior to the retina in the sub internal limiting membrane (ILM) space, in the sub hyaloid space or in the vitreous [17]. Among the above, sub ILM hemorrhage tends to remain localized, as the potential space beneath the ILM is limited. A sub ILM hemorrhage at the fovea obliterates the foveal contour on OCT [18]. Retinal veins may become dilated and tortuous and if associated with the widespread hemorrhages described above, anemic retinopathy may mimic CRVO [19], [20]. Besides the hemorrhages described; anemic retinopathy may also exhibit cotton wool spots, retinal edema and venous tortuosity [21]. 

In young patients who have no other recognizable risk factors for CRVO, hyperviscosity syndromes have to be ruled out. Evaluation of homocysteine, activated protein C resistance, protein C activity, protein S activity, antithrombin III activity, antiphospholipid antibodies, and anticardiolipin antibodies may be performed [22]. However, in our patient, the systemic malignancy is likely to be responsible for hyperviscosity and increased hematocrit. Elevated blood viscosity contributes to vein occlusion by influencing the blood coagulability. 

Visual function in patients with CRVO depends on the extent of macular involvement. If there were minimal macular edema without any hemorrhages in front of the macula, the patient would retain good vision. With extensive ischemia and macular edema as seen in this patient, vision is significantly reduced. Treatment is aimed at reducing the macular edema with use of intravitreal anti-vascular endothelial growth factor agents (Ranibizumab or Aflibercept) and treating the underlying systemic abnormality. 

In our patient, we postulate that rheological changes in blood due to breast carcinoma, anemia and hyperviscosity were responsible for the onset of CRVO.

## Notes

### Competing interests

The authors declare that they have no competing interests.
